# Supplementary material for: Direct observation of tensile-strain-induced nanoscale magnetic hardening
Source: Nat Commun. 2023 Jul 5;14:3963. doi: 10.1038/s41467-023-39650-8 (PMC10322833; doi:10.1038/s41467-023-39650-8)
Supplement: Supplementary file 1 — Supplementary Information [file 41467_2023_39650_MOESM1_ESM.pdf]

Supplementary Information for

Direct observation of tensile-strain-induced nanoscale magnetic hardening

Deli Kong[1,2], András Kovács[1]*, Michalis Charilaou[3], Fengshan Zheng[1,4], Lihua Wang[5], Xiaodong Han[5]* and Rafal E. Dunin-Borkowski[1]

[1] Ernst Ruska-Centre for Microscopy and Spectroscopy with Electrons and Peter Grünberg Institute, Forschungszentrum Jülich, Jülich, 52428, Germany.

[2] School of Physics and Optoelectronics, Faculty of Science, Beijing University of Technology, Beijing, 100124, China.

[3] Department of Physics, University of Louisiana at Lafayette, Lafayette, Louisiana 70504, United States.

[4] Spin-X Institute, School of Physics and Optoelectronics, South China University of Technology, Guangzhou, China.

[5] Institute of Microstructure and Properties of Advanced Materials, Beijing University of Technology, Beijing 100124, China.

*Corresponding authors Email: a.kovacs@fz-juelich.de, xdhan@bjut.edu.cn

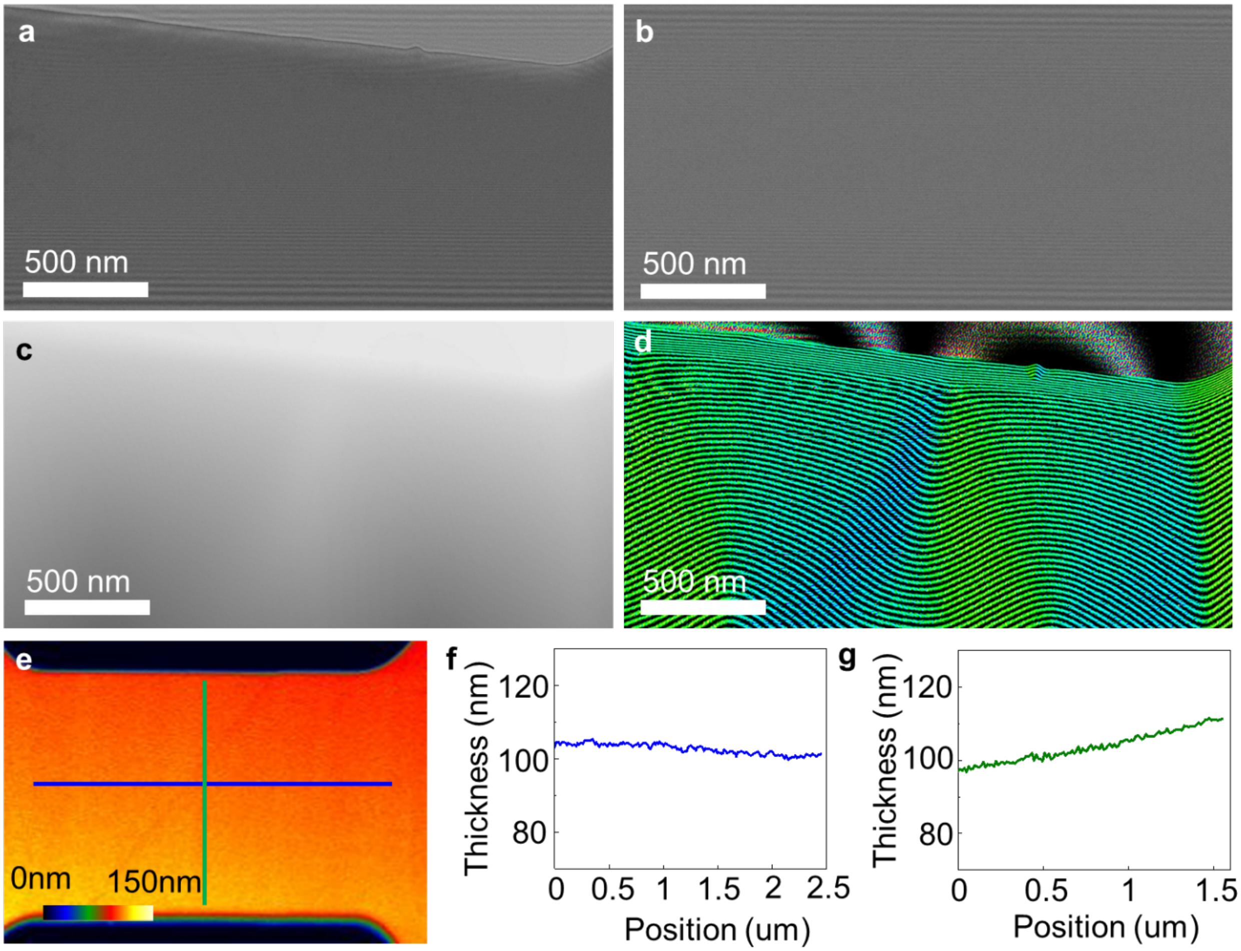

Supplementary Fig. 1. Processing of off-axis electron holograms and thickness measurement. **a** Off-axis electron hologram recorded from the Ni sample. **b** Reference hologram recorded from vacuum. **c** Extracted total phase shift map. **d** Projected in-plane magnetic induction map. The contour spacing is 2π/3 radians. The finely-spaced contour lines at the edge of the specimen are likely to be associated with an ion beam sputtering induced artifact. **e** Thickness map of the Ni sample extracted from a zero-loss peak intensity EELS measurement. Thickness variations along the blue (horizontal) and green (vertical) lines are plotted in **f** and **g**, respectively, showing a relatively uniform specimen thickness.

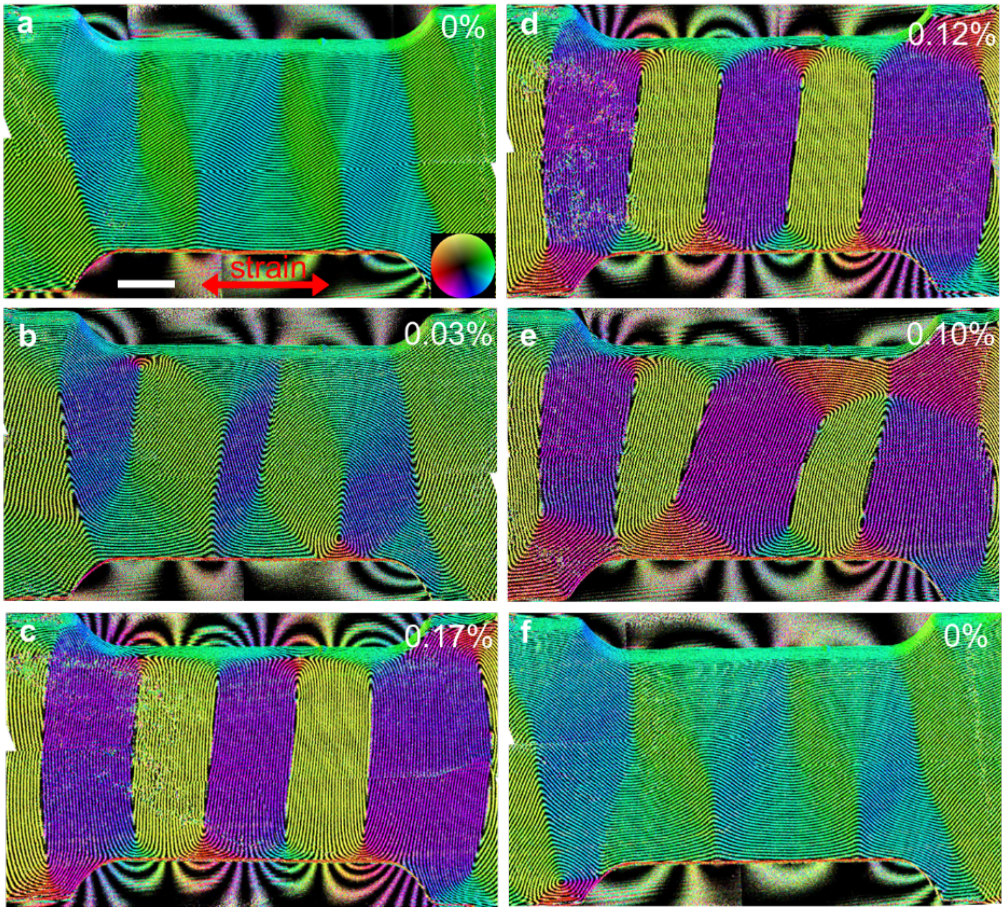

Supplementary Fig. 2. Magnetic induction maps recorded during the first tensile cycle. a - c Tensile process. **d - f** Release process. The colors indicate the projected in-plane magnetic field direction. The contour spacing is $2\pi/3$ radians. The strain is marked in the upper right corner of each figure. The scale bar is 500 nm. The first tensile cycle was used to measure the temperature range of the deformation device required to introduce magnetic state changes and to optimize the Fresnel defocus imaging and off-axis electron holography experiments (image and hologram acquisition, biprism voltage, magnification, holography set-up), while the straining process remained within the elastic regime. The second tensile cycle was then carried out using the optimized parameters in a systematic manner.

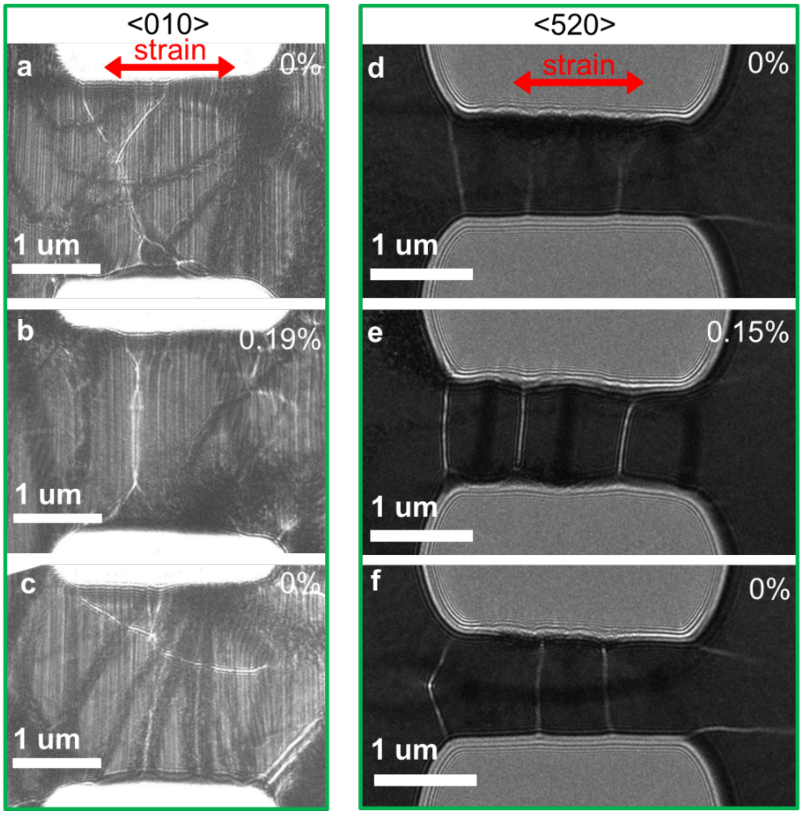

Supplementary Fig. 3. Magnetoelastic studies of Ni samples with different crystallographic orientations. **a** - **c** Tensile cycle in which the tensile direction is along the <010> zone axis direction of Ni, as marked with a red double-headed arrow. The defocus value is -1.8 mm. **a** The unstrained Ni contains random magnetic domain walls (bright lines). **b** The 180° magnetic domain wall turns orthogonal to the tensile direction with increasing strain. **c** The direction of the domain wall returns to a random orientation when the strain is released. **d** - **f** Another tensile cycle, in which the tensile direction is along the <520> zone axis direction of Ni. The defocus value is -2 mm. A similar behavior of the domain wall was observed with strain. The strain is marked in the upper right corner of each image.

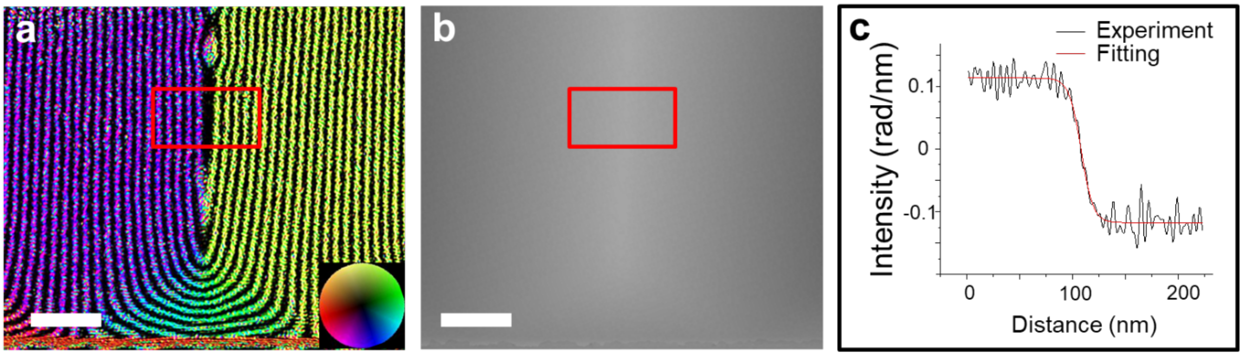

Supplementary Fig. 4. Example of 180° magnetic domain wall width measurement. **a** Magnetic induction map of the lamella. **b** Phase shift map. **c** Differential of the phase shift (black line) and non-linear curve fit (red line) to the function used to determine the domain wall width. The scale bar is 200 nm.

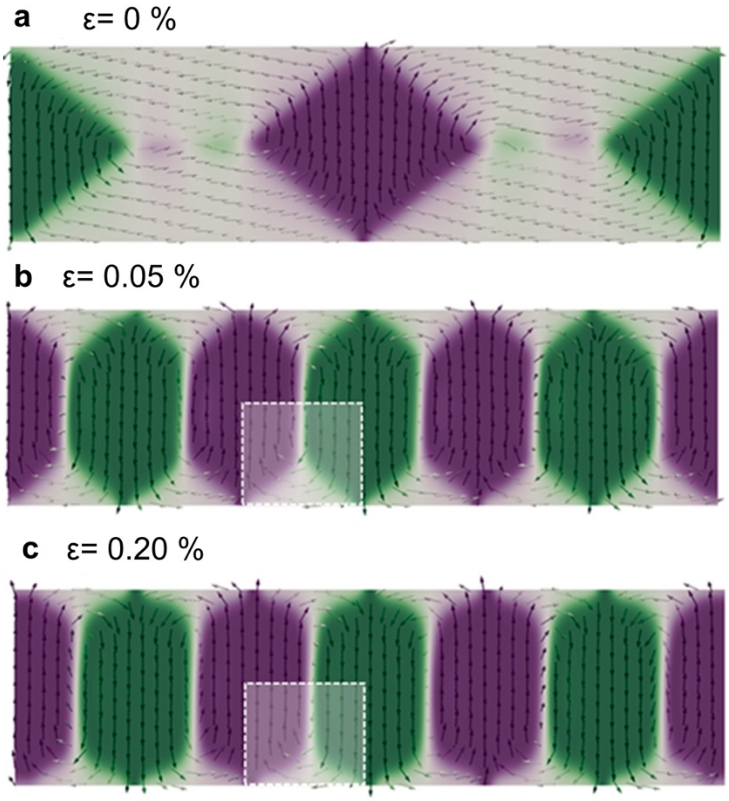

Supplementary Fig. 5. Simulated contour plots of magnetization in the Ni film at different strains. Equilibrium state of the magnetization obtained from micromagnetic simulations at (a) zero strain*, i.e.*, without strain-induced magnetic anisotropy, (b) at 0.05% strain and (c) at 0.20% strain, corresponding to 5 kJ/m^2 and 20 kJ/m^2 strain-induced magnetic anisotropy, respectively. With increasing strain, and in turn induced magnetic anisotropy, the 180º domain walls become increasingly sharp and the regions of magnetization rotation due to stray field closure close to the sample edges, as marked by the dashed-lined square, become smaller.

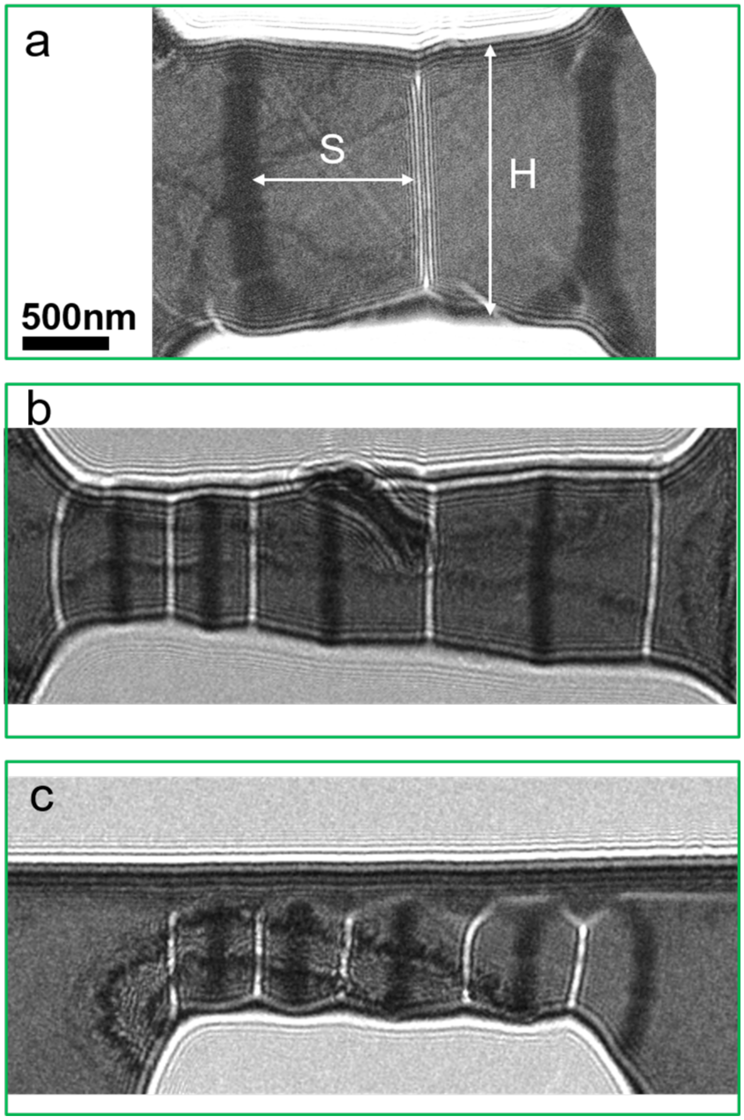

Supplementary Fig. 6. Example of variation in domain size with sample width. a - c show underfocus Fresnel images of Ni samples with different heights (H) during deformation processes. The defocus value used was 2 mm. The apparent difference in magnetic domain wall width between the images results from the different thicknesses of the specimens.

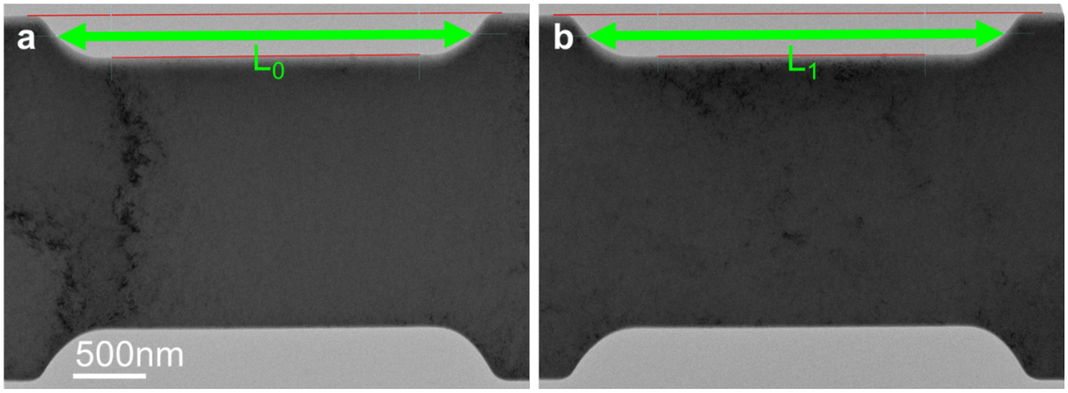

Supplementary Fig. 7. Example of strain measurement. a, **b** BF TEM images before and during the deformation process. First, two red lines tangent to the edge and root of the lamella were drawn as references. Second, the two distances were measured. The two red lines are not exactly parallel. A green line segment contacting the lamella root was then placed across the middle point of the two distances. The length of the green line segment was regarded as the length of the lamella. The lengths of lamella in (a) and (b) are 2.8536 µm (L_0) and 2.8594 µm (L_1), respectively. Thus, the strain is 0.20% according to the formula strain = $(L_1 - L_0)/L_0$. Since the length of one pixel in the image is 1.1 nm, the error in the strain is ±0.04 %.

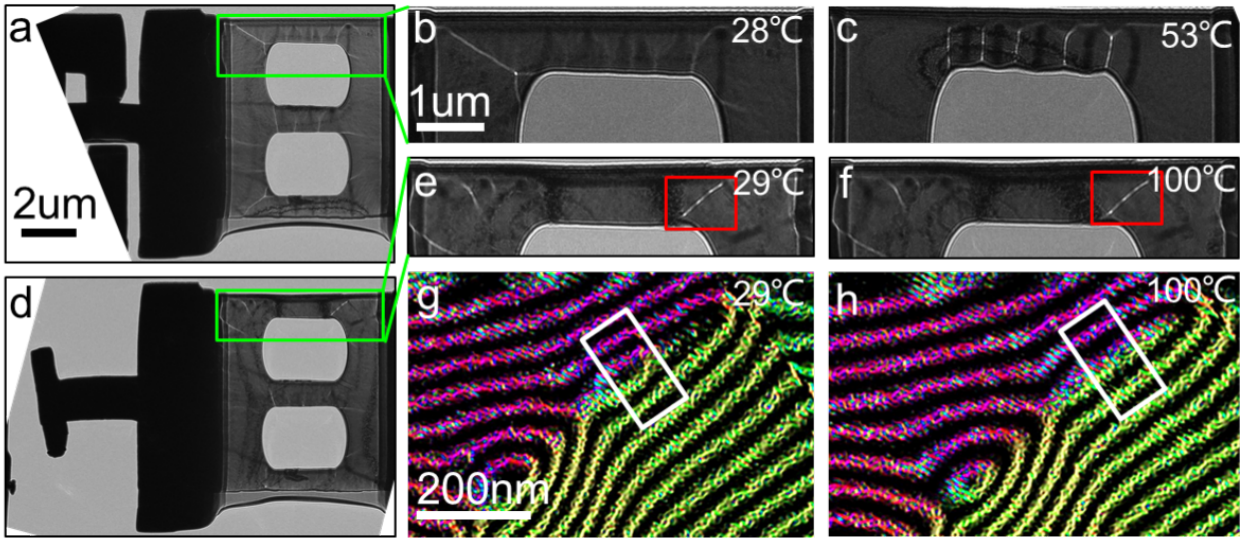

Supplementary Fig. 8. Impact of elevated temperature on the magnetic state of the Ni nanostructure. a Overview of the device and electron-transparent regions before straining. **b**, **c** Fresnel defocus images showing changes in magnetic structure of the green rectangular area in (a) during the tensile process. The experimental temperature was increased from 28 to 53 ℃. **d** Overview of the device after removing the hooks. **e**, **f** Fresnel defocus images of green rectangular area in (d) during the heating process. The magnetic state barely changes during the heating process. The temperature is marked at the upper right corner of each image. **g**, **h** Magnetic induction maps and recorded from the red rectangular areas marked in (e) and (f), respectively. The magnetic domain wall widths in the regions marked white rectangles in (g) and (h) are 53 nm and 49 nm, respectively.
