# Supplementary material for: Direct observation of tensile-strain-induced nanoscale magnetic hardening
Source: Nat Commun. 2023 Jul 5;14:3963. doi: 10.1038/s41467-023-39650-8 (PMC10322833; doi:10.1038/s41467-023-39650-8)
Supplement: Supplementary file 3 — Description of Additional Supplementary Files [file 41467_2023_39650_MOESM3_ESM.pdf]

Title: Supplementary Movie 1.
Description: Motion of magnetic domain walls during the 2nd tensile cycle. The movie comprises Fresnel defocus images captured during the 2nd straining cycle. The defocus value is -1.6 mm.

Title: Supplementary Movie 2.
Description: Motion of magnetic domain walls after stacking fault formation. The movie comprises Fresnel defocus images showing magnetic domain wall movement during a straining cycle after stacking fault formation in the Ni sample.

Title: Supplementary Movie 3.
Description: Control of magnetic structure by stress. By changing of the direction of the hook on the right side of the sample, the magnetization direction could be controlled in the lamella on the left side of the image. The scale bar is 2 µm.
